# A Single Nucleotide Polymorphism in the *Il17ra* Promoter Is Associated with Functional Severity of Ankylosing Spondylitis

**DOI:** 10.1371/journal.pone.0158905

**Published:** 2016-07-14

**Authors:** Jose Ramón Vidal-Castiñeira, Antonio López-Vázquez, Roberto Diaz-Peña, Paula Diaz-Bulnes, Pablo Martinez-Camblor, Eliecer Coto, Pablo Coto-Segura, Jacome Bruges-Armas, Jose Antonio Pinto, Francisco Jose Blanco, Alejandra Sánchez, Juan Mulero, Ruben Queiro, Carlos Lopez-Larrea

**Affiliations:** 1 Immunology Department, Hospital Universitario Central de Asturias, Oviedo, Spain; 2 Facultad de Ciencias de la Salud, Universidad Autónoma de Chile, Talca, Chile; 3 Department of Statistics, Hospital Universitario Central de Asturias, Oviedo, Spain; 4 Facultad de Ciencias de la Educación, Universidad Autónoma de Chile, Santiago de Chile, Chile; 5 Molecular Genetics Department, Hospital Universitario Central de Asturias, Oviedo, Spain; 6 Dermatology II Department, Hospital Universitario Central de Asturias, Oviedo, Spain; 7 Institute for Molecular and Cell Biology (IBMC), University of Porto, Porto, Portugal; 8 Rheumatology Service, Instituto de Investigación Biomédica de A Coruña (INIBIC), Complexo Hospitalario Universitario de A Coruña (CHUAC), Sergas, Universidade da Coruña (UDC), A Coruña, Spain; 9 Rheumatology Service, Puerta del Hierro University Hospital, Majadahonda, Madrid; 10 Rheumatology Service, Hospital Universitario Central de Asturias, Oviedo, Spain; 11 Fundación Renal Iñigo Álvarez de Toledo, Madrid, Spain; INSERM-Université Paris-Sud, FRANCE

## Abstract

The aim of this study was to identify new genetic variants associated with the severity of ankylosing spondylitis (AS). We sequenced the exome of eight patients diagnosed with AS, selected on the basis of the severity of their clinical parameters. We identified 27 variants in exons and regulatory regions. The contribution of candidate variants found to AS severity was validated by genotyping two Spanish cohorts consisting of 180 cases/300 controls and 419 cases/656 controls. Relationships of SNPs and clinical variables with the Bath Ankylosing Spondylitis Disease Activity and Functional Indices BASDAI and BASFI were analyzed. BASFI was standardized by adjusting for the duration of the disease since the appearance of the first symptoms. Refining the analysis of SNPs in the two cohorts, we found that the rs4819554 minor allele G in the promoter of the IL17RA gene was associated with AS (p<0.005). This variant was also associated with the BASFI score. Classifying AS patients by the severity of their functional status with respect to BASFI/disease duration of the 60th, 65th, 70th and 75th percentiles, we found the association increased from p60 to p75 (cohort 1: p<0.05 to p<0.01; cohort 2: p<0.01 to p<0.005). Our findings indicate a genetic role for the IL17/ILRA axis in the development of severe forms of AS.

## Introduction

Ankylosing spondylitis (AS) is a chronic inflammatory rheumatic disease that primarily involves the axial skeleton, whose susceptibility is clearly attributable to genetic factors [1, 2]. The high frequency of HLA–B27 in patients with spondylarthropathies such as AS (95% of patients with AS carry B27) has emerged as one of the best examples of a disease association with an HLA marker[3, 4]. The HLA–B27 family contains a large number of allelic variants or subtypes that differ in terms of ethnic distribution and whose heterogeneity has been previously determined in various populations[5]. However, population studies have indicated that only 2–5% of HLA–B27positive subjects develop the disease[6, 7]. These data suggest that this biomarker is clearly not sufficient on its own to cause disease, and it is evident that susceptibility to AS is affected by other environmental and genetic factors[8].

Recently, genome-wide association studies have shown that non-major histocompatibility complex (non-MHC) regions are involved in disease susceptibility[9–11], specifically genomic regions such as 1p, 2p, 2q, 3p, 9q, 10q, 11p, 16q, and 19q[12]. In fact, some studies have associated different variants of ERAP1 and IL23R and KIR genes with AS[13–16]. Despite the great advances stemming from the GWAS studies, some unexpected challenges also emerged[17, 18]. Genetic factors also influence disease prognosis and clinical outcome, but little is known about this association.

The functional severity, radiographic severity and activity of the AS, respectively measured with the Bath Ankylosing Spondylitis Functional Index (BASFI), the Bath Ankylosing Spondylitis Radiology Index (BASRI) and the Bath Ankylosing Spondylitis Disease Activity Index (BASDAI), can help us study the pathogenesis of the disease. Recently, several studies have associated some biomarkers with the functional and radiographic severity status of the patient[19, 20] and with their BASDAI score[21].

Thus, the aim of this study was to determine whether common and rare DNA variants in the exome regions and in the promoters are associated with the risk of developing AS or have an effect on disease severity. Exome sequencing was used for these purposes in a group of patients with advanced disease status. It is a powerful tool that can help us identify rare genetic traits that affect disease evolution. We extend the exome sequencing to promoter regions, identifying minor variants as possible biomarkers associated with disease severity.

## Patients and Methods

### Study population

Eight AS patients were selected for exome sequencing on the basis of severe clinical parameters (mean BASFI, 6.8 ± 1.1; mean BASDAI, 6.4 ± 1.8). These patients had severe pain along the spine and/or in the pelvis, sacroiliac joints, heels and chest. The high degree of joint damage made it difficult for them to do their daily activities. For validation purposes, two Spanish cohorts of patients (S1 Table) and healthy controls were also selected. Cohort 1 comprised 180 patients with AS and 300 healthy control subjects, recruited from the *Hospital Universitario Central de Asturias* (Oviedo, Spain) and the *Hospital Universitario A Coruña* (A Coruña, Spain).For the replication phase (Cohort 2), 419 patients with AS and 656 healthy controls were recruited from the *Hospital Universitario Puerta de Hierro* (Madrid, Spain), which is a participant institution in the Spanish National Spondyloarthropathies Registry (REGISPONSER) (Table 1). There were 599 unrelated patients with AS (mean age, 50.3 ±10.5 years; 78.3% men) and 956 healthy controls (mean age, 52.0 ±16.0 years; 59% men). All patients were diagnosed in Rheumatology Units in accordance with the Modified New York Criteria and had at least 10 years of follow-up from the first symptoms of the disease. The disease was defined as severe or non-severe according to the BASDAI and the BASFI.

**Table 1:** A) IL17RA rs4819554 distribution in cohort 1. Statistical significance (p<0.05) was lost when a Bonferroni correction was applied. B) IL17RA rs4819554 distribution in cohort 2.

**A) Cohort 1**				
**Allele**	**Patients (2n = 360)**	**Controls (2n = 600)**	**p**_**c**_	**OR (95% CI)**
A	273 (75.8)	486 (81)	NS	-
G	87 (24.2)	114 (19)		
**Genotype**	**Patients (n = 180)**	**Controls (n = 300)**	**p (AA vs. Gx)**	**OR (95% CI)**
AA	104 (57.8)	194 (64.7)	NS	-
AG	65 (36.1)	98 (32.7)		
GG	11 (6.1)	8 (2.7)		
**B) Cohort 2**				
**Allele**	**Patients (2n = 838)**	**Controls (2n = 1312)**	**p**	**OR (95% CI)**
A	639 (76.3)	1052 (80.2)	<0.05	1.26 (1.02–1.55)
G	199 (23.7)	260 (19.8)		
**Genotype**	**Patients (n = 419)**	**Controls (n = 656)**	**p (AA vs. Gx)**	**OR (95% CI)**
AA	240 (57.3)	416 (63.4)	<0.05	1.29 (1.01–1.66)
AG	159 (37.9)	220 (33.5)		
GG	20 (4.8)	20 (3)		

**Cohort 1:** From the Hospital Universitario Central de Asturias (Oviedo, Spain) and the Hospital Universitario A Coruña (A Coruña, Spain).

**Cohort 2:** From the Hospital Universitario Puerta de Hierro (Madrid, Spain) which participated in the Spanish National Spondyloarthropathies Registry (REGISPONSER).

Notes: p_c_, Bonferroni-adjusted probability.

Hardy-Weinberg equilibrium: p>0.05

The study was granted ethical approval by the Regional Ethics Committee for Clinical Investigation of the participating hospitals (Regional Ethics Committee of Clinical Research of *Principado de Asturias*; Ethics Committee of Clinical Research of Galicia; Regional Ethics Committee of Clinical Research of Madrid) and conducted in accordance with the Declaration of Helsinki. All patients and control subjects gave their written informed consent before enrolling in the study.

### Whole-exome sequencing and analysis

Whole-exome sequencing was performed in eight AS patients with severe forms of the disease using SureSelect Target Enrichment Human All Exon + UTR kit (71mb) (Agilent Technologies) compatible with the SOLID platform. We evaluated the quality and quantity of DNA patients by agarose gel electrophoresis, measuring absorbance at 260 nm withNanoDrop1000 and quantifying the results by fluorescence Qubit.

The quality and quantity of the libraries obtained were evaluated by Qubit and an Agilent 2100 Bioanalyzer. The libraries were subjected to a process of emulsion PCR for clonal amplification fragments, followed by enrichment and chemical modification to allow loading in the reaction chamber, employing the protocols and recommendations provided by Life Technologies for sequencing with the SOLiD 5500XL system.

The quality and quantity of the microspheres obtained from the libraries were estimated taking into account the parameters derived from the work-flow analysis (WFA). The reactions for obtaining sequences of 75nt+25 nt (paired-end) were subsequently carried out in a SOLiD 5500XL system. Data quality was assessed from the parameters estimated by the sequencing software.

Bioscope version 1.3 was used to map and pair the reads to the NCBI human genome reference (hg19, GRCh37). Single nucleotide variants (SNVs) and indels were selected for subsequent analysis by the following four criteria. First, all SNPs selected were new variants that had not previously been associated with AS. Also all the selected variants resulted in a non-synonymous aminoacid change in the protein that was able to modify the functionality based on a predictive model. The SNPs on the promoter should be present in regions for transcription factor binding also based on a predictive model. Second, the MAF differences of each SNP should be greater than 20% between selected patients and the general population. Third, the depth of coverage of the exome sequencing should be greater than 30. Finally, these SNPs should be included in genes of immunological relevance or that encode proteins associated with osteogenesis and/or cellular adhesion. We also searched for previously unreported protein-altering variants that occurred in more than one subject and in the promoter.

SNVs and indels were called using Samtools-0.1.18[22], Picard tools-1.67 and the Genome Analysis Toolkit (GATK)[23].

### SNV validation

57 significant SNVs obtained from the exome sequencing analysis were reanalyzed by Sanger sequencing.

### Single nucleotide polymorphism genotyping

The SNVs confirmed by Sanger sequencing were analyzed in the two cohorts of patients and controls. These variants were genotyped by amplifying the region containing the polymorphic site and hybridization with fluorescent-labeled probes in an RT-PCR based on TaqMan^®^ SNP Genotyping Assays (Applied Biosystems). Genomic DNA from patients and healthy donors was extracted from peripheral blood with the Magtration-MagaZorb DNA Common Kit-200 N using the Magtration 12GC system (Precision System Science Co., Ltd., Woerrstadt, Germany) and the Maxwell^®^ 16 Blood Purification Kit using the Maxwell^®^ 16 Instrument (Promega Corporation, Madison, Wisconsin, USA).

### Statistical analysis

The χ^2^ test was used to examine differences in genotype and allele frequencies between the groups and to determine deviations from the Hardy-Weinberg equilibrium. Odds ratios (ORs) and their 95% confidence intervals (CIs) were also calculated. Student’s *t* test for independent samples was used to compare differences in means between the groups. Standard linear regression was used to relate disease severity measures (BASFI and BASDAI) with the genotype. The association between AS functional severity and the presence or absence of the IL17RA SNP was assessed using the BASFI index. To standardize the measure of functional severity, we corrected the BASFI for the duration of AS, from the onset of the disease, taken as the appearance of the first symptoms, until the time of the last BASFI and BASDAI score measure. The average time of evolution from disease onset was 10 years. We performed three association analyses using the χ^2^ test, each with different criteria for defining the functionally severe group. Based on the clinical features, we classified the patients in the top 60^th^ (p60), 65^th^ (p65), 70^th^ (p70) or 75^th^ (p75) percentile of BASFI/disease duration in the severe group[13]. The magnitude of association was expressed as an OR and its 95% CI.

## Results

### Exome sequencing analysis

Based on the aforementioned criteria, we established which SNPs were of potential interest in this study. We selected 57 SNPs that we then validated by Sanger sequencing in order to exclude false positives. This left 27 SNPs, all of which were present in genes associated with inflammation processes, bone and cartilage synthesis, and misfolded protein or other processes (S2 Table). The SNPs analyzed were all exon missense variants or promoter variants. A test validation of the deviation from Hardy-Weinberg equilibrium was performed for each SNP.

### IL17RA rs4819554 polymorphism analysis

Refining these SNPs, we found that the rs4819554 located in the IL17RA promoter region was significantly associated with AS in cohort 1 (minor allele G, p<0.05; OR = 1.36), but not when a Bonferroni correction was applied (Table 1). Nevertheless, when we analyzed this SNP in cohort 2 it was clearly associated with AS (minor allele G, p<0.05; OR = 1.26) (Table 1). When the two cohorts were combined this allele was also more frequent in patients than in the control group, (p<0.005; OR = 1.29) (data not shown).

**Fig 1:**
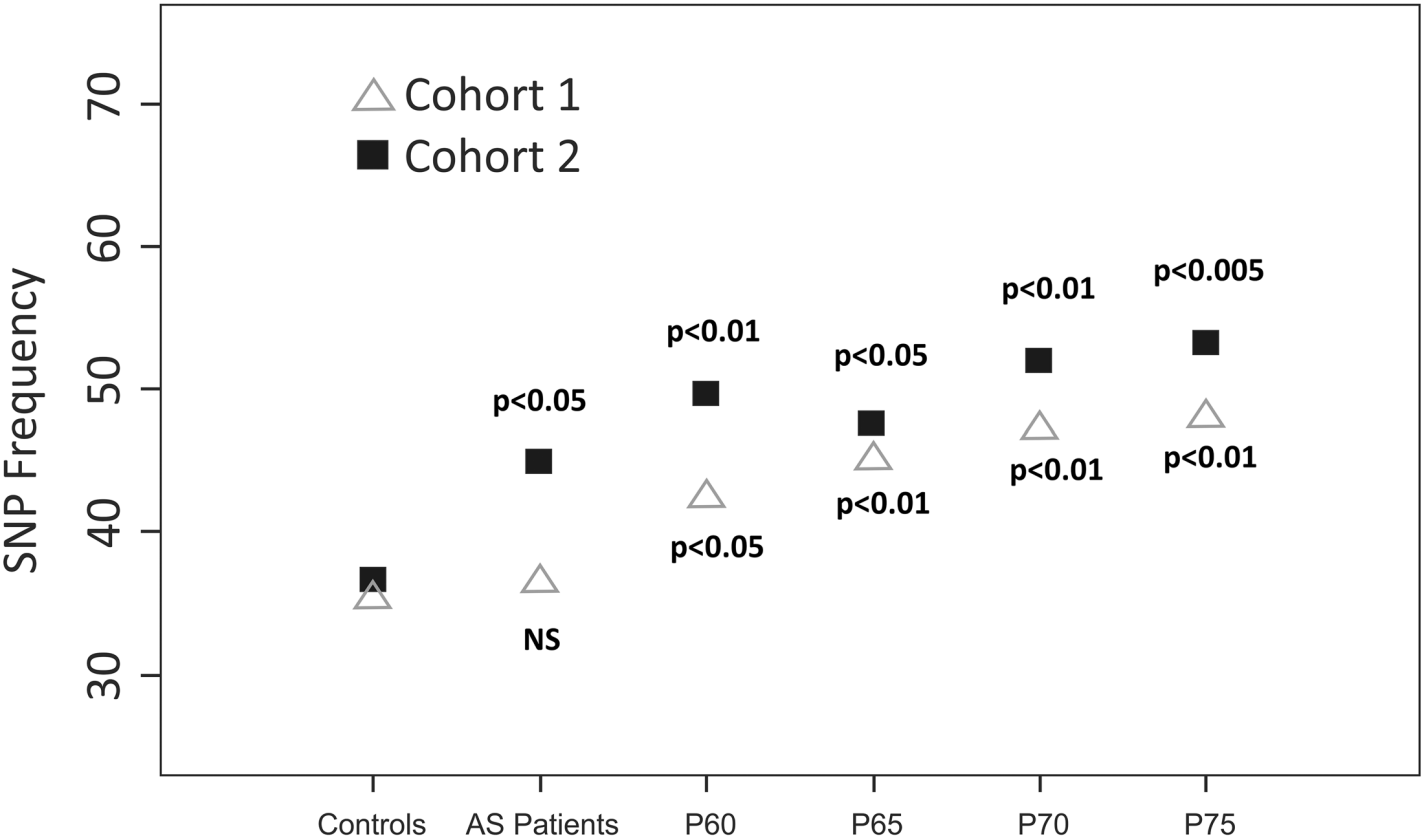
IL17RA rs4819554 genotype frequency (Gx) based on BASFI/t-duration. Note: **Cohort 1.** (BASFI/t p75 *vs*. controls: 48.0% *vs*. 35.3%, p<0.01). R^2^ = 65%. **Cohort 2.** (BASFI/t p75 *vs*. controls: 53.2% *vs*. 36.6%, p<0.005) R^2^ = 95%.

Subsequently, we analyzed the association of rs4819554 SNP with the BASFI and BASDAI and found that the presence of this SNP was associated with the severity of AS (Table 2). The BASFI score was higher in patients with the Gx genotype (p<0.01) and the association persisted after correcting for the duration of disease evolution (p<0.01). These patterns were also found in cohort 2. However, the BASDAI score only had a statistically significant association in the second cohort (p<0.05), including when we adjusted the score for the duration of disease evolution (p<0.05). We then corrected the BASFI association for the duration of AS since the onset of the disease [BASFI/t]. A representative of the SNP rs4819554 association with [BASFI/t] is shown in Fig 1. The most highly significant association was with p75 (Cohort 1, p<0.01; Cohort 2, p<0.005).

**Table 2:** Clinical characteristics related to disease severity with respect to the rs4819554 genotype distribution.

	Cohort 1 (n = 180)			Cohort 2 (n = 419)			Both cohorts (n = 599)		
	AA genotype n = 104	Gx genotype n = 76	p	AA genotype n = 240	Gx genotype n = 179	p	AA genotype n = 344	Gx genotype n = 255	p
BASDAI, mean (SD)	3 (2.2)	3.3 (2.2)	NS	3.9 (2.5)	4.4 (2.3)	<0.05	3.7 (2.7)	4.1 (2.6)	<0.05
BASDAI adjusted by duration of disease evolution, mean (SD)	0.19 (0.29)	0.23 (0.35)	NS	0.18 (0.12)	0.22 (0.13)	<0.05	0.19 (0.24)	0.22 (0.29)	NS
BASFI, mean (SD)	3.1 (2.6)	4.2 (2.7)	<0.01	3.7 (2.7)	4.3 (2.8)	<0.05	3.7 (2.7)	4.2 (2.8)	<0.05
BASFI adjusted by duration of disease evolution, mean (SD)	0.15 (0.13)	0.21 (0.14)	<0.01	0.15 (0.12)	0.19 (0.13)	<0.01	0.15 (1.4)	0.19 (1.4)	<0.01

Note: Gx genotype includes GG and GA genotypes

In conclusion, the rs4819554 SNP was found to be associated with patients with poor evolution, as determined by the BASFI score.

## Discussion

Several genome-wide association studies (GWASs) have described many SNPs in the genome that are associated with AS susceptibility[11, 24]. Although some studies have related various gene variants to radiographic and functional severity in AS[19, 20], to our knowledge the present study is the first to use exome sequencing to examine the association of allelic variants with the functional severity of the patient, as measured by the BASFI.

We performed whole-exome sequencing plus promoter regions in a subset of severe AS patients to identify novel and minor variants contributing to AS risk. After sequencing, we found that the rs4819554 G minor allele, located in the promoter of IL17RA gene, was associated with AS in the two clinical cohorts. This variant has recently been related to alopecia areata susceptibility[25] and psoriasis[26], and we had found it to be responsible for impaired renal function[27]. Conversely, this SNP was not previously associated to AS in GWAS probably due to a different point of view in patient selection. We have incised in clinical characteristics, as previously described in Patients and Methods section. Moreover, we observed that the rs4819554 polymorphism can influence the severity of the functional status of AS patients, measured as BASFI/t duration and BASDAI/t duration scores. These findings suggest that the rs4819554 may be a good biomarker of the severity of the disease, and that it could be applied early in the course of AS in patients identified as having a potentially severe functional outcome.

The IL-17 family comprises a group of cytokines made up of six members (IL-17A to F) and five receptors (IL-17RA to E) with an active role in the acute and chronic immune responses[28, 29]. The prototypical member of the IL17 family, IL-17A, defines a new subset of CD4+ effector T (Th17) cells and this cytokine induces the production of inflammatory mediators from epithelial and endothelial cells and fibroblasts[30]. In relation to AS, the levels of IL17A are higher in the sera and synovial fluid of patients[31, 32], and a high frequency of Th17 cells has been found in the peripheral blood, synovial fluid and inflamed bone marrow of patients with AS, implying a role for these cells in inflammation in AS[33, 34].

The IL17RA receptor might be crucial to the majority of IL17 family members[35]. This receptor is expressed in many types of cells, including epithelial cells, fibroblasts, B and T lymphocytes, myelomonocytic cells and marrow stromal cells[29]. Several common polymorphisms in the genes of the IL-17 family have been linked to the risk of developing cancer, and autoimmune and inflammatory diseases[36–38]. However, none of these studies evaluated the rs4819554 SNP in IL-17RA.

The rs4819554 SNP is located in the promoter region of IL17RA and could thus have a functional effect by inducing or inhibiting the transcription of the gene. Sequencing analysis revealed other promoter polymorphisms in linkage disequilibrium with rs4819554 that were predicted to affect the binding of transcription factors (TFs), such as those of the Ikaros (IK) family. This family of TFs is involved in regulating Th17 cell differentiation through the promotion of Th17 lineage-determining genes and in repressing the expression of genes that limit the development of Th17[39]. A putative effect of these promoter polymorphisms on TF binding could also explain the reported differences in IL17RA protein expression and the amount of mRNA in CD14+ peripheral blood monocytes between individuals with different genotypes[39].

Interleukin (IL)-23 is considered to be a central cytokine that acts in AS to regulate IL17 production. It is known from a murine model of AS that IL-23+ CD3+/CD4-/CD8- resident T cells are able to release IL-17, which is important for driving spinal inflammation. Levels of IL17A have been found to be higher in the sera and synovial fluid of AS patients[32]. Th17 cells are particularly abundant in the peripheral blood, synovial fluid and inflamed bone marrow of patients with AS, suggesting that they have a role in inflammation in AS. Several other cell types (especially Type 3 innate immune cells) defined as Lyn^−^IL-23R^+^NKp44^+^Tbet^+^RORc^**−**^ are also important sources for IL-17 production in AS. Other polymorphisms of the IL-23/IL-17A axis, such as SNPs in TRAF3IP2 that code for the adaptor Act1, a downstream target of IL-17R, which confers susceptibility to psoriatic arthritis, may be evaluated in AS[40]. Further studies to determine the effect of rs4819554 on gene expression would be of particular interest.

In conclusion, we report an association between the rs4819554 SNP in the promoter region of *IL17RA* and the risk of AS, with a stronger association in individuals with severe AS with respect to the BASFI score and the duration of the disease. Our results need to be validated in other populations, and other studies are required to measure the levels of circulating IL17RA in blood and synovial fluid and to confirm our results concerning the effect of this polymorphism on gene expression. The study of new biomarkers in relation to AS progression could help us develop a predictive algorithm based on the treatment regimen.

## Supporting Information

S1 Table Patients characteristics of two cohorts studied. (DOCX)

S2 Table Single nucleotide polymorphisms obtained from whole exome sequencing and confirmed by Sanger sequencing. (DOCX)
