# Supplementary material for: A Single Nucleotide Polymorphism in the Il17ra Promoter Is Associated with Functional Severity of Ankylosing Spondylitis
Source: PLoS One. 2016 Jul 14;11(7):e0158905. doi: 10.1371/journal.pone.0158905 (PMC4945092; doi:10.1371/journal.pone.0158905)
Supplement: S1 Table — (DOCX) [file pone.0158905.s001.docx]

**S1 Table: Patients characteristics of two cohorts studied.**

| Characteristics | Cohort 1  (n=180) | Cohort 2  (n=419) |
| --- | --- | --- |
| Gender n (%)  Male  Female | 150 (83.3)  30 (16.7) | 319 (76.1)  100 (23.9) |
| Age, mean (SD) | 49.8 (10.4) | 50.8 (10.4) |
| HLA-B27, n (%) | 173 (96.1) | 357 (85.2) |
| Time of evolution from first symptoms, years, mean (SD) | 20.1 (10) | 25.1 (10.1) |
| Age at first symptoms, years, mean (SD) | 23.9 (9) | 25.9 (9) |
| Age at disease diagnosis, years, mean (SD) | 36.5 (11.3) | 34.5 (11.4) |
|  |  |  |
| BASFI | 4.2 (2.7) | 4 (2.8) |
| BASDAI | 3.3 (2.2) | 4.2 (2.3) |
