# Supplementary material for: A Single Nucleotide Polymorphism in the Il17ra Promoter Is Associated with Functional Severity of Ankylosing Spondylitis
Source: PLoS One. 2016 Jul 14;11(7):e0158905. doi: 10.1371/journal.pone.0158905 (PMC4945092; doi:10.1371/journal.pone.0158905)
Supplement: S2 Table — (DOCX) [file pone.0158905.s002.docx]

**S2 Table: Single nucleotide polymorphisms obtained from whole exome sequencing and confirmed by Sanger sequencing.**

| GENE | Chromosome | Position | WT | Mut. | Minor allele frequency in patients | Minor allele frequency in population | Polymorphism | AA Position | WT AA | Mut. AA | TRANSCRIPT |
| --- | --- | --- | --- | --- | --- | --- | --- | --- | --- | --- | --- |
| EVC2 | 4 | 5690902 | T | C | 0.625 | 0.21 | rs4689278 | 230 | S | G | ENST00000344408 |
| POU5F1B | 8 | 128428823 | G | C | 0.875 | 0.52 | rs7002225 | 238 | E | Q | ENST00000465342 |
| COL6A5 | 3 | 130162395 | G | A | 1 | 0,18 | rs9883988 | 2188 | Q | R | ENST00000312481 |
| COL6A5 | 3 | 130174334 | A | G | 1 | 0.1 | rs819085 | 2205 | G | D | ENST00000312481 |
| TNFRSF10D | 8 | 23002090 | C | T | 0.25 | 0,04 | rs55636833 | 276 | R | H | ENST00000312584 |
| MPO | 17 | 56348106 | T | C | 0.25 | 0,02 | rs2759 | 749 | I | V | ENST00000340482 |
| IL17F | 6 | 52101844 | T | C | 0.375 | 0,05 | rs2397084 | 162 | E | G | ENST00000336123 |
| CARD8 | 19 | 48715196 | T | C | 0.25 | 0,03 | rs34632751 | 462 | Q | R | ENST00000391898 |
| ITGAM | 16 | 31289396 | T | C | 0.5 | 0,15 | rs1186125 | 441 | M | T | ENST00000544665 |
| ASCC2 | 22 | 30200713 | G | A | 0.25 | 0,04 | rs36571 | 423 | P | S | ENST00000307790 |
| PPP2R3A | 3 | 135721781 | C | G | 0.5 | 0,04 | rs34901937 | 481 | P | A | ENST00000264977 |
| PPP2R3A | 3 | 135720540 | A | G | 0.875 | 0,13 | rs9814557 | 67 | D | G | ENST00000264977 |
| TNFRSF10A | 8 | 23060256 | T | C | 0.75 | 0.45 | rs17620 | 141 | H | R | ENST00000221132 |
| SERPINA9 | 14 | 94936110 | G | A | 0.5 | 0,05 | rs45438398 | 41 | P | L | ENST00000337425 |
| IL7R | 5 | 35876274 | A | G | 0.75 | 0,23 | rs3194051 | 356 | I | V | ENST00000303115 |
| TNFRSF10D | 8 | 23002090 | C | T | 0.25 | 0,04 | rs55636833 | 276 | R | H | ENST00000312584 |
| NLRP12 | 19 | 54313707 | G | C | 0.375 | 0,04 | rs34971363 | 402 | F | L | ENST00000391773 |
| IL17F | 6 | 52101844 | T | C | 0.375 | 0,04 | rs2397084 | 162 | E | G | ENST00000336123 |
| ASCC2 | 22 | 30221120 | C | T | 0.25 | 0,07 | rs11549795 | 123 | V | I | ENST00000307790 |
| PPP2R3A | 3 | 135722264 | A | G | 1 | 0,15 | rs17197552 | 642 | S | G | ENST00000264977 |
| KIF24 | 9 | 34257095 | G | A | 0.875 | 0,14 | rs41274041 | 837 | S | F | ENST00000402558 |
| KIF24 | 9 | 34311020 | T | C | 0.75 | 0,07 | rs41274845 | 109 | R | G | ENST00000402558 |
| LCA5L | 21 | 40778182 | C | T | 0.625 | 0,06 | rs11558767 | 547 | G | S | ENST00000288350 |
| PRRC2B | 9 | 134334588 | A | G | 0.625 | 0,06 | rs34553878 | 417 | M | V | ENST00000357304 |
| DAPL1 | 2 | 159663616 | G | A | 0.75 | 0.03 | rs12535 | 66 | A | T | ENST00000409042 |
| CD200R1L | 3 | 112545910 | GT | G | 0.375 | 0.16 | rs58161637 | 203 | H | Q | ENST00000398214 |
| IL17RA | 22 | 17084145 | A | G | 0.75 | 0.23 | rs4819554**^1^** | - | - | - | - |

**^1^This polymorphism is included in the promoter.**
